# Dose-reduced [^18^F]PSMA-1007 PET is feasible for functional imaging of the renal cortex

**DOI:** 10.1186/s40658-021-00419-x

**Published:** 2021-10-29

**Authors:** Kristian Valind, Jonas Jögi, David Minarik, Gustav Brolin, Elin Trägårdh

**Affiliations:** 1 Wallenberg Centre for Molecular Medicine, Lund University, Lund, Sweden; 2 Clinical Physiology and Nuclear Medicine, Skåne University Hospital, Lund and Malmö, Sweden; 3 Radiation Physics, Skåne University Hospital, Lund and Malmö, Sweden

**Keywords:** PET-CT, PSMA, Renal function, Renal cortex, DMSA

## Abstract

**Background:**

In Prostate-specific membrane antigen (PSMA) positron emission tomography with computed tomography (PET-CT), there is significant renal uptake. The standard in renal cortical functional imaging is scintigraphy with technetium-99m labeled dimercaptosuccinic acid (DMSA). Using [^68^Ga]Ga-PSMA-11 PET for renal imaging has been suggested, but using [^18^F]PSMA-1007 has not been explored. The aims of this study were to establish the optimal time point for renal imaging after [^18^F]PSMA-1007 injection, to investigate the reproducibility of split renal uptake measurements, and to determine the margin for reduction in administered activity.

**Methods:**

Twelve adult male patients with prostate cancer underwent [^18^F]PSMA-1007 PET-CT at 8 time points up to 5.5 h post-injection (p.i.). List-mode data were binned to durations of 10 to 120 s per bed position (bp). Left renal percentage of total renal uptake (LRU%) was measured, and the difference between highest and lowest measurement per patient (“delta max”) was calculated. Images acquired at 1 h, 2 h, and 5.5 h p.i. with durations of 10 to 120 s/bp were rated regarding image quality.

**Results:**

Imaging at 2 h p.i. with 60 s/bp yielded acceptable quality in all cases. Increasing acquisition time to 15 min for a single bp would allow reducing administered activity to 0.27 MBq/kg, resulting in an effective dose of 0.4 mSv for a 1-year old child weighing 10 kg. The median delta max of LRU% measurements was 2.7% (range 1.8–7.3%).

**Conclusions:**

Renal [^18^F]PSMA-1007 PET-CT is feasible, with imaging 2 h p.i., acceptable split renal uptake variability, and effective dose and acquisition time comparable to those of [^99m^Tc]Tc-DMSA scintigraphy.

## Background

Prostate-specific membrane antigen (PSMA) has become the main target for positron emission tomography (PET) imaging in prostate cancer. PSMA PET imaging is combined with computed tomography (CT) into hybrid PET-CT studies that are used for initial staging and the detection of recurrence. However, PSMA is expressed in several normal tissues in addition to prostate cancer cells, including the renal proximal tubules [1]. Accordingly, studies on [^68^Ga]Ga-PSMA-11 and the more recently introduced [^18^F]PSMA-1007 have shown significant uptake in the renal parenchyma [2, 3]. The use of PSMA PET has been suggested for functional imaging of the renal cortex [4, 5]. [^68^Ga]Ga-PSMA-11 PET has been compared to technetium-99m labeled dimercaptosuccinic acid (DMSA) scan in two adult patients with pyelonephritis [6, 7]. A recent study has shown correlation between renal uptake of [^68^Ga]Ga-PSMA-11 and renal function as measured with glomerular filtration rate (GFR) [8]. A comparison of split renal function using technetium-99m labeled mercaptotriglycene ([^99m^Tc]Tc-MAG3) renography and [^68^Ga]Ga-PSMA-11 PET has shown reasonable agreement between the modalities [9].

Flourine-18 has theoretical advantages over gallium-68 for PET imaging, such as lower positron energy, which results in a higher spatial resolution [10]. Additionally, [^18^F]PSMA-1007 has lower renal clearance than [^68^Ga]Ga-PSMA-11 [2, 3], making it a promising candidate for renal parenchymal imaging.

[^99m^Tc]Tc-DMSA scintigraphy is a well-established method for functional imaging of the renal cortex. This method provides estimates of split renal function and images that are read for defects of tracer uptake [11, 12]. [^99m^Tc]Tc-DMSA scintigraphy is primarily used for the detection of localized parenchymal impairment or damage after pyelonephritis in children, so it is usually performed using significantly lower radiation doses than PSMA PET-CT [13].

To determine the feasibility of using [^18^F]PSMA-1007 PET for renal parenchymal imaging analogous to [^99m^Tc]Tc-DMSA scintigraphy, an imaging protocol for this specific use needs to be established, with a radiation burden that is on par with that received from a [^99m^Tc]Tc-DMSA scan. Therefore, the aims of this study were to establish the optimal time point for image acquisition after radiotracer injection, to investigate the variability of split renal uptake percentages, and to determine the margin for reduction in administered activity while retaining sufficient image quality.

## Methods

### Patients

The study included 12 adult male patients (Table 1) who were referred to the Department of Clinical Physiology and Nuclear Medicine, Skåne University Hospital, Malmö, Sweden, for clinical [^18^F]PSMA-1007 PET-CT. Patients were referred due to prostate cancer, either newly diagnosed intermediate to high-risk disease or biochemical recurrence after treatment. Inclusion was made as part of a study on the biokinetics and dosimetry of [^18^F]PSMA-1007, with acquired data being used for the current study as well. The study was approved by the Swedish Ethical Review Authority (#2020–00,689) and was performed in accordance with the Declaration of Helsinki. All patients gave written informed consent prior to partaking in the study.

**Table 1:** Patient characteristics

Parameter	Mean ± SD (range)
Age	67 ± 7.3 (53 − 77)
BMI	25.3 ± 3.1 (21.3 − 30.1)
GFR	84.7 ± 19.5 (54.8 − 112.6)

BMI: body mass index. GFR: glomerular filtration rate

#### PET-CT

Patients were injected with 4.0 ± 0.4 MBq/kg of [^18^F]PSMA-1007 (range 3.4–5.1) and underwent PET-CT imaging from the mid-thigh to the vertex of the skull using two GE Discovery MI PET-CT systems (GE Healthcare, Milwaukee, WI, USA). While whole-body imaging was performed, only the parts covering the kidneys were evaluated as part of this study. Repeated PET-CT imaging was performed at 4 time points within 0.5 h post injection (p.i.), and further images were then acquired at 1 h, 2 h, 3.5 h, and finally at 5.5 h p.i. In total, 8 scans per patient were obtained. The first 4 scans per patient had an acquisition time of 30 s/bed position (bp). At 1 h, 2 h, and 3.5 h p.i. images were acquired at 120 s/bp, while images at 5.5 h p.i. had an acquisition time of 180 s/bp. List-mode acquisition was used for all PET imaging. The list mode data from scans performed at least 0.5 h p.i. were binned into series with shorter acquisition times (10 and 20 s/bp for the 0.5 h p.i. data; 10, 20, 30, 60, and 90 s/bp for the > 0.5 h p.i. data; and additionally, 120 s/bp for the 5.5 h p.i. images). Due to a low image quality in the kidney region, determined visually, no rebinning of the original 30 s/bp images from the first 3 acquisitions into series with shorter acquisition times was performed.

Reconstruction was performed using the block-sequential regularization expectation maximization algorithm (BSREM), marketed as Q.Clear (GE Healthcare, Milwaukee, WI, USA), using a β-value of 800 [14]. Kidneys were manually segmented in the PET images using the RECOMIA platform [15]. Segmentations were made with images displayed using an inverse gray scale, with a range from standardized uptake value (SUV) 0 to the highest SUV in the scanned volume. Acquisitions were segmented individually based on the original data, with these segmentations being used for all rebinned durations as well. For time points below 0.5 h p.i., the 0.5 h p.i. segmentations were used, as the patient did not move between these acquisitions. This approach was chosen as segmenting each duration individually would introduce another source of variability. One patient had significant excreted activity in the renal pelvises, which was excluded from segmentation. Segmentation was performed by a single reader.

### Uptake/activity quantification

For all reconstructed images, the activity in each segmented renal volume of interest (VOI) was measured in MBq. The percentage of activity in the left renal VOI of the total renal activity (left renal uptake percentage—LRU%) was calculated for each acquisition time point and duration. The variability of these measurements was determined by calculating the standard deviation (SD) and difference between the highest and lowest LRU% measurements (“delta max”) for each patient. The activity concentration of both renal VOIs was calculated for each image acquisition.

To be able to determine the concentration of [^18^F]PSMA-1007 in renal tissue, with implications for the selection of acquisition time points for evaluation, venous blood samples were drawn before the injection of [^18^F]PSMA-1007 and immediately after each image acquisition. The activity concentration in 2-mL whole-blood samples was measured using a gamma counter (HIDEX AMG, Hidex Oy, Turku, Finland). The renal volume was assumed to consist of 25% blood and 75% renal tissue, and the renal tissue activity concentration was calculated using the following equation:

$${{C}_{tissue}=C}_{kidney}\cdot \left(\frac{{V}_{kidney}}{{V}_{tissue}}\right)-{C}_{blood}\cdot \left(\frac{{V}_{blood}}{{V}_{tissue}}\right)$$

$${{C}_{tissue}=C}_{kidney}\cdot \left(\frac{1}{0.75}\right)-{C}_{blood}\cdot \left(\frac{0.25}{0.75}\right)$$

### Visual evaluation of image quality

Three independent observers rated images that were acquired at 1 h, 2 h, and 5.5 h p.i. with acquisition times of 10 to 120 s. The 1 h p.i. series was chosen because it had the highest renal tissue activity concentration in most patients (9 of the 12 patients). The series acquired at 2 h p.i. was chosen since this time point is the most common for imaging in PSMA PET. The 5.5 h p.i. images were chosen to test the far limits of image quality and the time between injection and acquisition. Observers used a 5-point Likert scale to rate the overall image quality (1: very inadequate; 2: inadequate; 3: acceptable; 4: good; 5: very good). The ratings of quality encompassed noise, contrast, and perceivable level of detail.

### Dosimetry

The effective dose from [^18^F]PSMA-1007 for an adult has been calculated to be 0.022 mSv/MBq [3], but no calculation of the effective dose to pediatric patients has been performed. To assess the effective dose for a pediatric patient, we multiplied the adult dose with a factor derived from the quotient between the adult dose and the dose for a one-year-old child for six other fluorine-18 labeled radiopharmaceuticals: fluorodeoxyglucose ([^18^F]FDG), fluoro-dihydroxyphenylalanine ([^18^F]FDOPA), [^18^F]fluorocholine, flutemetamol, fluroethyltyrosine ([^18^F]FET), and flourothymidine ([^18^F]FLT) [16–20].

### Statistical analysis

The LRU% data were tested for normal distribution using the Shapiro–Wilk test. To compensate for variability in LRU% between patients when examining measurement reproducibility, the LRU% measurements were normalized against the median measurement in each patient. We sought to determine whether acquisition time point or duration had a significant effect on measurements of split renal uptake. To this end, the normalized LRU% measurements were tested for their dependence on the acquisition duration and time point p.i. using Kruskal–Wallis rank sum tests. Post-hoc analysis was performed using Dunn’s test, with Bonferroni correction for multiple comparisons. Statistical analyses were performed using the R statistical software package (Version 4.0.5; The R Foundation for Statistical Computing, Vienna, Austria).

## Results

For 2 patients, list mode files for the 2 h p.i. time points were lost, leaving 362 series available in the analysis of split renal function and 206 series in the visual analysis of image quality.

### Time points for imaging

**Fig. 1:**
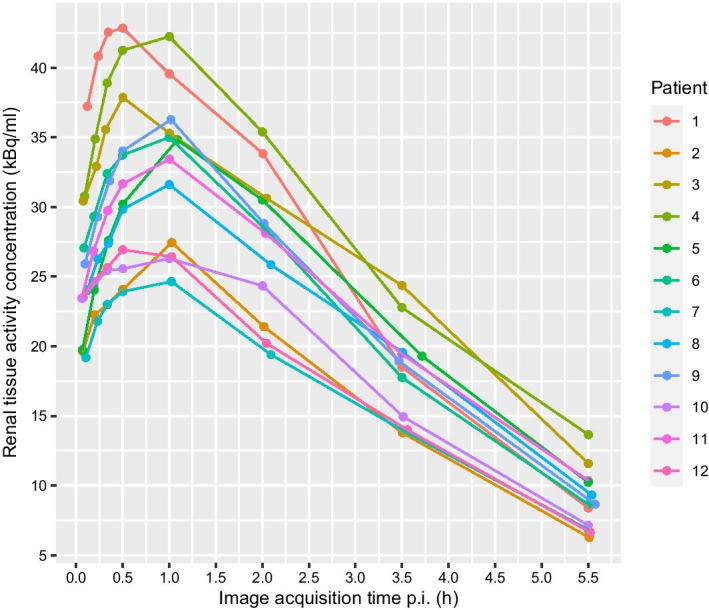
Renal tissue activity concentration at different time points after ^18^F-PSMA-1007 injection

**Fig. 2:**
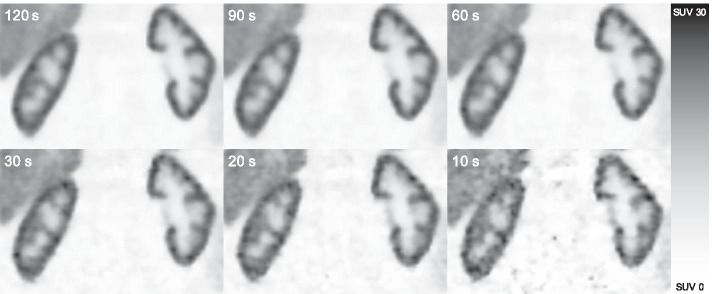
Coronary images of the kidneys acquired at 2 h p.i. in a representative patient showing image quality at different acquisition durations per bp. Reducing acquisition duration results in more noise, which is visible, especially in the 20-s and 10-s images. bp: bed position, p.i.: post-injection

**Fig. 3:**
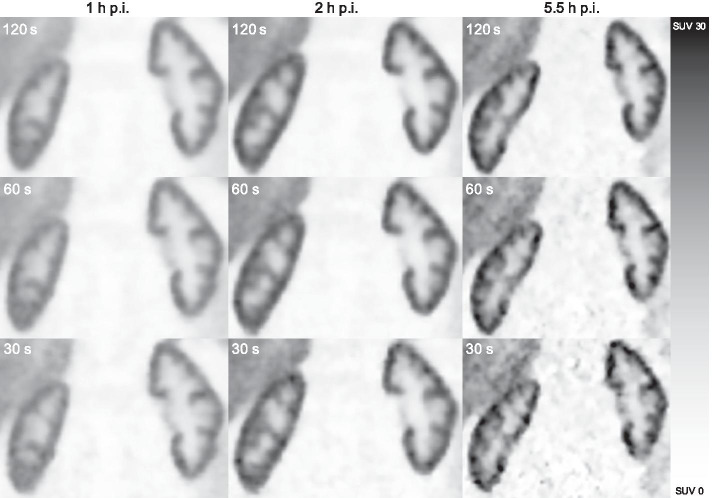
Coronary images of the kidneys in a representative patient showing image quality at different acquisition durations per bp (rows) and time points after injection (columns). bp: bed position, p.i.: post-injection

For 9 of the 12 patients, the highest renal tissue activity concentration was measured at the 1-h p.i. time point, with the 0.5-h p.i. time point having the highest renal tissue activity concentration in three patients (Fig. 1). Figures 2 and 3 show representative images for different acquisition durations and time points after injection.

### Split renal uptake

**Fig. 4:**
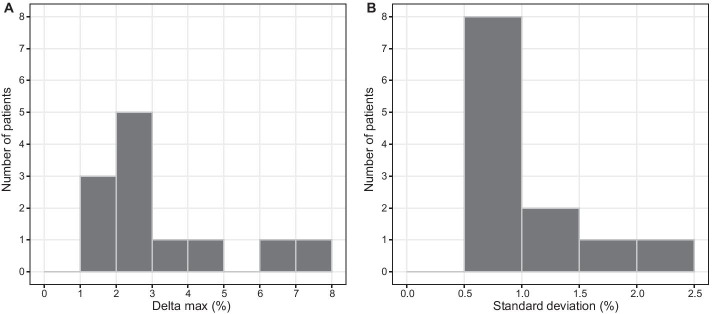
Histograms of (**a**) difference between highest and lowest measured left renal uptake percentage (LRU%) within a patient (“delta max”) and (**b**) standard deviation of LRU% measurements

**Fig. 5:**
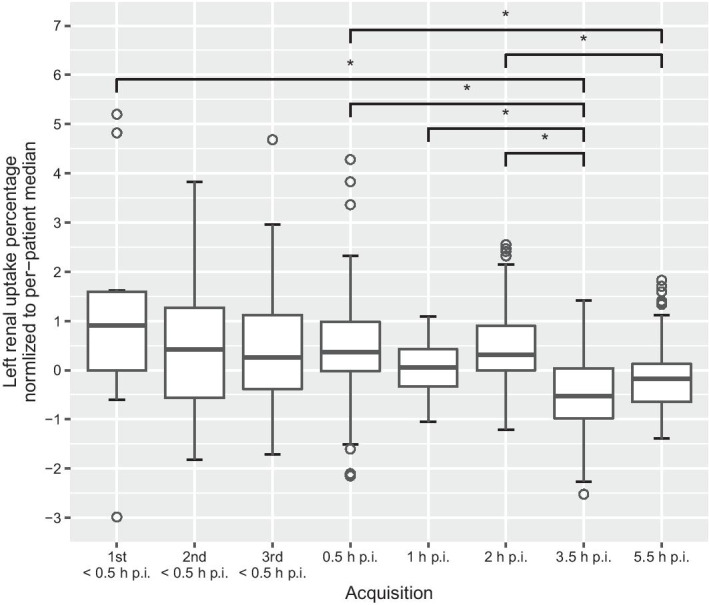
Left renal uptake percentages (LRU%) normalized to per-patient median to allow comparison between acquisitions for all patients. The central line in each box represents the median. The lower and upper bounds of each box represent the 25^th^ and 75^th^ percentiles, respectively. Bars represent 1.5 times the interquartile ranges, with circles representing outliers outside these. The 3.5-h p.i. acquisition had significantly lower LRU% than the 1st acquisition and the 0.5 h, 1–h, and 2-h p.i. acquisitions. The 5.5-h acquisition had significantly lower LRU% than the 0.5- h and 2-h p.i. acquisitions. No other significant differences were found. * *p* < 0.005 using Dunn’s test with Bonferroni correction for multiple comparisons. p.i.: post-injection

The measurements of LRU% ranged from 4.9% to 55.4% (median 48.3%). The maximum difference between measurements of LRU% in a single patient (“delta max”) ranged from 1.8% to 7.3% (median 2.7%) (Table 2, Fig. 4). The standard deviation of LRU% measurements per patient was between 0.51 and 2.2 (median 0.76; Fig. 4). Both the original and median-normalized data failed the Shapiro–Wilk test (*p* < 0.0001 for both), meaning that a normal distribution could not be assumed for the measurements of LRU%. Thus, nonparametric statistical methods were used for further analysis. The Kruskal–Wallis rank sum tests performed on the normalized LRU% measurements revealed a significant dependence on the acquisition duration (*p* = 0.0017) and on acquisition time p.i. (*p* < 0.0001). Post hoc analysis using Dunn’s test with Bonferroni correction showed no significant differences between any combinations of acquisition durations. Significant differences were found when comparing the acquisitions at 3.5 h p.i. and 5.5 h p.i. to other acquisitions (Fig. 5). For the non-normalized LRU% measurements, Kruskal–Wallis rank sum testing revealed no dependence on the acquisition time p.i. (*p* = 0.2375) or acquisition duration (*p* = 0.8890).

**Table 2:** Left renal uptake percentages

Patient	Median ± SD	Range	Delta max
1	46.7 ± 1.1	44.6 − 48.5	4.0
2	46.3 ± 2.2	43.8 − 51.2	7.3
3	54.8 ± 0.6	53.5 − 55.4	1.9
4	48.3 ± 0.8	46.9 − 49.8	2.9
5	47.7 ± 0.5	46.9 − 48.6	1.8
6	51.1 ± 1.1	48.1 − 52.1	4.0
7	51.1 ± 0.6	49.8 − 52.6	2.8
8	5.8 ± 1.6	4.9 − 11.0	6.1
9	47.3 ± 0.7	46.3 − 48.9	2.6
10	48.9 ± 0.8	47.8 − 50.5	2.7
11	51.6 ± 0.6	50.4 − 52.7	2.3
12	46.6 ± 0.5	45.6 − 47.6	1.9

Delta max is the result of subtracting the lowest measurement per patient from the highest

### Image quality

**Fig. 6:**
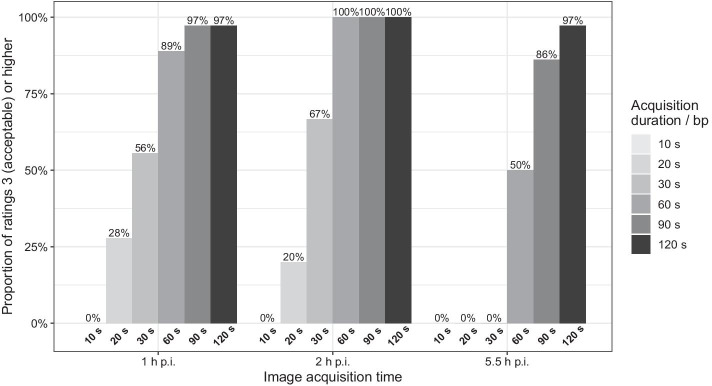
Percentage of all ratings ≥ 3 (acceptable) per acquisition time p.i. and acquisition duration per bp. bp: bed position, p.i.: post-injection

Counting each reviewer’s score individually, $\ge$ 95% acceptable or better ratings ($\ge$ 3 out of 5) were achieved for images acquired at 1 h, 2 h, and 5.5 h p.i. with acquisition durations of least 90 s/bp, 60 s/bp, and 120 s/bp, respectively (Fig. 6). All reviewers gave at least acceptable ratings to all images from the 2-h p.i. time point with acquisition durations of $\ge$ 60 s/bp.

### Dosimetry

According to Giesel et al., the effective dose to an adult is 0.022 mSv/MBq [3]. Using a conversion factor from an adult to a one-year-old of 4.9, the effective dose to the one-year-old is 0.11 mSv/MBq. The conversion factor was calculated as the mean of 5.0, 4.0, 5.0, 4.5, 5.9, and 5.1, based on the effective doses of [^18^F]FDG, [^18^F]FDOPA, [^18^F]fluorocholine, flutemetamol, [^18^F]FLT, and [^18^F]FET, respectively [16–20]. Assuming that only a single bed position is needed, prolonging PET acquisition from 60 s to 15 min would allow for a 15-fold reduction of the administered activity while retaining the same image quality. An acquisition time of 15 min is not uncommon in [^99m^Tc]Tc-DMSA imaging [11]. This imaging protocol assumes a similar biodistribution of [^18^F]PSMA-1007 in 1-year-old as in adult males. A reduction from 4.0 MBq/kg to 0.27 MBq/kg would thus result in an effective dose of 0.3 mSv for a one-year-old child weighing 10 kg. Adding a short abdominal CT-scan for attenuation correction, which on a GE Discovery MI system can be done with as little as 0.1 mSv, would bring the total effective dose to 0.4 mSv.

## Discussion

Imaging at 2 h after injection of 4 MBq/kg [^18^F]PSMA-1007 with an acquisition duration of 60 s/bp yielded acceptable or better image quality for evaluation of the kidneys in 100% of cases. The median difference between the highest and lowest measurements of LRU% was 2.7%. These results show that [^18^F]PSMA-1007 PET can be used for measurement of split renal uptake with reasonable precision and that there is ample room for reduction in injected activity without compromising image quality.

For a one-year-old patient weighing 10 kg, a specialized 15 min [^18^F]PSMA-1007 PET-CT protocol would result in an effective dose of 0.4 mSv. It may be possible to further lower the effective dose by omitting the low dose CT, if attenuation correction is not required. The effective dose would be 0.67 mSv to the same patient from a [^99m^Tc]Tc-DMSA scintigraphy using 18 MBq according to the EANM pediatric dosage card [13, 17]. Using our protocol, acquisition of high resolution tomographic renal images is thus possible with similar effective dose and acquisition time as those of a planar [^99m^Tc]Tc-DMSA scintigraphy. One potential disadvantage of using [^18^F]PSMA-1007 over [^99m^Tc]Tc-DMSA is the comparatively high uptake in liver and spleen, which may hinder automatic segmentation of the kidneys.

The variability of the LRU% measurements is comparable to what has been published for [^99m^Tc]Tc-DMSA scintigraphy [21], although we are not aware of any publication on [^99m^Tc]Tc-DMSA studies with repeated imaging after a single injection to determine split function measurement stability.

A delta max above 5% was observed for the LRU% in two patients. One of these patients had considerable asymmetry of renal uptake, which reduces the precision of such measurements [22]. The images of the other patient with a high delta max indicated varying degrees of respiration-induced artifacts that affected the liver and right kidney. Considering this, some measure of respiratory gating or motion correction could perhaps improve the accuracy of split renal uptake measurements.

After per-patient normalization, LRU% measurements were significantly lower in the last two acquisitions when compared to earlier ones. We interpret this as primarily being an effect of measurement imprecision and a low number of patients. A larger study, preferably with a reference standard, would be able to elucidate whether the acquisition time point has a clinically relevant effect on split uptake measurements. The large span of LRU% measurements may explain why no significant differences were found in the non-normalized data.

### Limitations

All patients included in the study were male and above 50 years of age, whereas patients referred for clinical [^99m^Tc]Tc-DMSA scintigraphy are mainly children with a more equal distribution between sexes. Our proposed protocol assumes a similar biodistribution of [^18^F]PSMA-1007 in pediatric patients and adult males, which to our knowledge has not been established. This assumption is based on the similarity of [^99m^Tc]Tc-DMSA biodistribution across age groups, but will have to be investigated further. This study did not examine the effects of a prolonged acquisition on image quality, such as from the introduction of motion induced artifacts. Furthermore, this study did not include a structured reading of the images with regard to the presence of uptake defects. Further study of the mechanisms linking [^18^F]PSMA-1007 uptake to renal function is needed before clinical use can be recommended.

## Conclusion

Functional imaging of the renal cortex is feasible using a [^18^F]PSMA-1007 PET-CT protocol with effective dose and acquisition time that are comparable to those of [^99m^Tc]Tc-DMSA scintigraphy. Imaging at the 2-h p.i. time point provides the best image quality for this purpose. The variability of measurements of the split renal uptake percentages is sufficiently low for clinical use.

## Data Availability

The data used in this study are available from the corresponding author on reasonable request.

## Abbreviations

bp: Bed position
BMI: Body mass index
BSREM: Block-sequential regularization expectation maximization
CT: Computed tomography
DMSA: Dimercaptosuccinic acid
FDG: Fluorodeoxyglucose
FET: Fluroethyltyrosine
FDOPA: Fluoro-dihydroxyphenylalanine
FLT: Flourothymidine
GFR: Glomerular filtration rate
MAG3: Mercaptotriglycene
LRU%: Left renal uptake percentage
PET: Positron emission tomography
p.i.: Post-injection
PSMA: Prostate-specific membrane antigen
SD: Standard deviation
SUV: Standardized uptake value
VOI: Volume of interest
